# Functionality of membrane proteins overexpressed and purified from *E. coli* is highly dependent upon the strain

**DOI:** 10.1038/s41598-019-39382-0

**Published:** 2019-02-25

**Authors:** Khadija Mathieu, Waqas Javed, Sylvain Vallet, Christian Lesterlin, Marie-Pierre Candusso, Feng Ding, Xiaohong Nancy Xu, Christine Ebel, Jean-Michel Jault, Cédric Orelle

**Affiliations:** 1 Université de Lyon, CNRS, UMR 5086 “Molecular Microbiology and Structural Biochemistry”, IBCP, 69367 Lyon, France; 2 Department of Chemistry & Biochemistry, Old Dominion University, Norfolk, VA 23529 USA; 3 Université Grenoble Alpes, CNRS, CEA, IBS, 38000 Grenoble, France

## Abstract

Overexpression of correctly folded membrane proteins is a fundamental prerequisite for functional and structural studies. One of the most commonly used expression systems for the production of membrane proteins is *Escherichia coli*. While misfolded proteins typically aggregate and form inclusions bodies, membrane proteins that are addressed to the membrane and extractable by detergents are generally assumed to be properly folded. Accordingly, GFP fusion strategy is often used as a fluorescent proxy to monitor their expression and folding quality. Here we investigated the functionality of two different multidrug ABC transporters, the homodimer BmrA from *Bacillus subtilis* and the heterodimer PatA/PatB from *Streptococcus pneumoniae*, when produced in several *E. coli* strains with T7 expression system. Strikingly, while strong expression in the membrane of several strains could be achieved, we observed drastic differences in the functionality of these proteins. Moreover, we observed a general trend in which mild detergents mainly extract the population of active transporters, whereas a harsher detergent like Fos-choline 12 could solubilize transporters irrespective of their functionality. Our results suggest that the amount of T7 RNA polymerase transcripts may indirectly but notably impact the structure and activity of overexpressed membrane proteins, and advise caution when using GFP fusion strategy.

## Introduction

Membrane proteins account for about 20–30% of synthesized proteins in all organisms^1^. They play key roles in human diseases and are targeted by more than half of therapeutic drugs. Native membrane proteins are generally insufficiently abundant to isolate material for biochemical and structural studies. Therefore, membrane proteins are often overexpressed in heterologous systems. The bacterium *Escherichia coli* is the most convenient and widely used system for overexpression of both soluble and membrane proteins^2,3^. The reason is largely historical due to a wealth of knowledge regarding its physiology, the availability of effective genetic tools and well-known advantages: (i) easy DNA transformation; (ii) fast growth and high cell density cultures; (iii) inexpensive culture costs and (iv) high yield of overexpression. The *E. coli* BL21(DE3) strain together with T7 promoter-based plasmids have been extensively employed to massively overexpress proteins. In this system, the T7 RNA polymerase gene is located in the DE3 prophage of the chromosome under the control of the IPTG-inducible L8-UV5 *lac* promoter, which is a more powerful variant of the wild-type lac promoter. Two base pair substitutions make the −10 promoter sequence closer to the consensus one recognized by bacterial sigma factors, thereby recruiting the RNA polymerase more effectively and decreasing its dependence on CAP/cAMP stimulation for full activation. A third mutation, located in the CAP/cAMP binding site, decreases the affinity for CAP/cAMP. These 3 mutations thus create a stronger promoter that is less sensitive to catabolic repression^4^. The BL21(DE3) strain is also deficient in Lon and OmpT proteases, and the T7 RNA polymerase transcribes ~8 times faster than native *E. coli* RNA polymerases^5^ to generate high level of mRNA available for protein synthesis. However, such strategy may not be the most appropriate for some proteins, especially those that are toxic. As such, the overexpression of membrane proteins may have detrimental effects by their intrinsic function, improper folding or by exceeding the capabilities of the machineries involved in membrane protein biogenesis and protein secretion^6–8^. Twenty years ago, Miroux and Walker designed a simple screening approach to isolate BL21(DE3) mutant strains displaying improved membrane protein overexpression abilities^9^. BL21(DE3) cells expressing toxic membrane proteins were plated on medium containing the IPTG inducer to select for surviving colonies that can cope with the toxic effects associated with membrane protein overexpression. With this approach, the C41(DE3) and C43(DE3) strains were selected and are now widely used to overexpress membrane proteins, although they do not improve yields for all of them. Much later, it was discovered that the C41(DE3) and C43(DE3) contained 3 mutations in the lacUV5 promoter^10^. The two mutations in the -10 region turned back the lacUV5 promoter into the much weaker wild-type lac promoter. Moreover, in contrast to the lacUV5 promoter, the wild-type lac promoter is susceptible to catabolite regulation. Therefore, a reduced transcription rate in these derivative strains likely explains why overexpression of many membrane proteins is hardly toxic for their growth and results in substantially improved membrane protein overexpression yields. Although the identified genetic differences lie in the lacUV5 promoter, they may have indirect effects on mRNA stability, protein translation and folding or stress response. For instance, Wagner and colleagues hypothesized that high transcription levels of membrane proteins is counterproductive because it leads to the saturation of the Sec translocon^10^. As a result, most overexpressed and endogenous membrane proteins fail to insert into the membrane and aggregate, resulting in cellular deleterious effects. Based on these observations, this team successfully engineered a BL21(DE3) variant strain named Lemo21(DE3), in which the activity of the T7 RNA polymerase can be finely tuned by its natural inhibitor T7 lysozyme, whose gene is under the control of the rhamnose promoter^10,11^. The optimization of membrane protein synthesis and their insertion into the membrane can thus strongly minimize the toxic effects associated with membrane protein overexpression, resulting in higher bacterial biomass and protein yield. However, it is unclear whether the quality of membrane proteins successfully incorporated into the membrane can also be affected by the T7 polymerase expression. The most popular tool for quality control of membrane proteins remains the use of GFP fusion to monitor the level of fluorescence produced by the host, which was shown to directly correlate with the amount of protein of interest properly inserted in the cytoplasmic membrane^12^.

Here, we report the overexpression of two different multidrug ABC transporters in various *E. coli* expression strains governed by T7 RNA polymerase/promoter system. The first one is the heterodimeric PatA/PatB transporter from *Streptococcus pneumoniae*. Although its biochemical characterization is at early stages^13,14^, its implication in clinical resistance to antibiotics was clearly demonstrated by the group of Piddock^15–17^. The second one is the homodimeric BmrA transporter from *Bacillus subtilis*^18^. BmrA is a fairly robust membrane protein, which can be produced and purified from *E. coli* in high yields for mechanistic characterization^19,20^, detergent/reconstitution method developments^21–24^ and structural studies, i.e. cryo-EM^25^ or solid-state NMR^26^. Here, we show that these membrane proteins can be expressed and addressed to the membrane equally well in different *E. coli* strains, but they are substantially more functional and easier to solubilize by mild detergents when overexpressed in the C41(DE3) strain. In addition, we also analyzed a BmrA-GFP chimeric construct since such a strategy has been widely used to screen for the production and structural integrity of membrane proteins, based on the assumption that a fluorescent GFP is a direct readout of the proper folding of the fused protein of interest. Overall, our data strongly suggest that membrane proteins may be targeted to *E. coli* membranes but not necessarily in a properly folded form. Our study illustrates that functionality of membrane proteins drastically depends on the *E. coli* expressing strain and cautions against choosing an expression strain based solely on expression levels. Moreover, the fluorescence of the fused GFP will not necessarily attest of the quality of the membrane protein of interest and this assay should thus be used with caution.

## Results

### Overexpression of PatA/PatB in various *E. coli* strains

**Figure 1:**
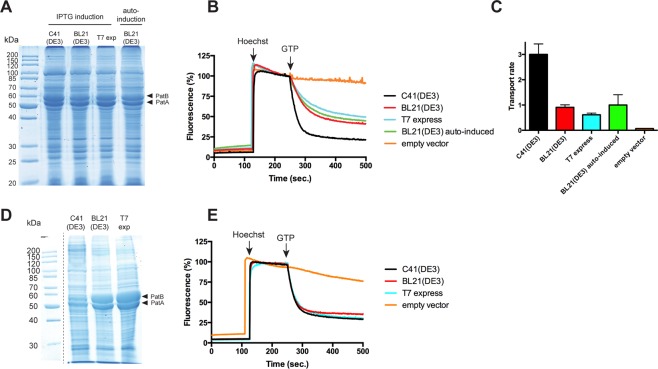
Overexpression of PatA/PatB in various *E. coli* strains and functionality of the transporter. (**A**) Expression of the transporter was either induced overnight by IPTG when cells reached OD_600_~1.8 or by auto-induction. Membrane fractions were analyzed by 12% SDS-PAGE stained with Coomassie blue. Twenty µg of proteins were loaded in each lane. (**B**) Hoechst 33342 transport assays with inverted membrane vesicles prepared from the various strains in panel A. 50 µg of total membrane proteins were used for each sample. (**C**) Hoechst 33342 transport rates were measured by initial slopes following GTP addition. The empty vector was used in the C41(DE3) strain. The data represent the average of two independent experiments, each with triplicates, and error bars indicate the standard deviation. (**D**) Expression of the transporter was induced by IPTG in exponential phase for 5 h. Membrane fractions were analyzed by 12% SDS-PAGE stained with Coomassie blue. Twenty µg of proteins were loaded in each lane. Dash line delineates cropped images from different parts of the same gel. (**E**) Hoechst 33342 transport assays with inverted membrane vesicles prepared from the various strains in panel (D). The empty vector was used in the C41(DE3) strain. Two hundred µg of total membrane proteins were used for each assay. Data shown are representative of two independent experiments.

PatA/PatB is a heterodimeric multidrug ABC transporter from *Streptococcus pneumoniae*^13,27,28^ involved in clinical resistance to fluoroquinolones. We overexpressed this transporter in different *E. coli* strains: BL21(DE3), C41(DE3), and T7 express. The latter is a BL21 derivative in which the T7 polymerase gene is inserted in the lactose operon and is thus expressed under the control of the lac promoter. This design permits controlled induction of the polymerase and consequently, inducible control of transcription of genes downstream of the T7 promoter. This system provides potential advantages over strains that carry the T7 RNA polymerase on a lysogenic prophage DE3. Although λDE3 is normally dormant in the host chromosome, the expression of toxic proteins may results in the induction of the SOS cascade that directly or indirectly damage the *E. coli* chromosome leading to cell lysis. The expression of PatA/PatB was induced overnight at 25 °C by IPTG addition at high OD_600 nm_ (~1.7), as published before^13,14^. In addition, the BL21(DE3) strain was either induced by IPTG or by using an auto-induction media. Auto-induction under diauxic growth conditions in media containing glucose, lactose, and glycerol is a convenient approach for *lac*-based expression systems in *E. coli*. It relies on inducer exclusion between glucose and lactose, preventing the induction by lactose before glucose consumption^29^. In these conditions, similar amounts of the transporter were overexpressed in the membrane fractions of all strains (Figs 1A and S1 for empty vector control). These results indicate that the transporter could be efficiently addressed to the *E. coli* membrane regardless of the strains employed.

### Drug transport catalyzed by PatA/PatB in inverted membrane vesicles

Exceeding the capacity of the bacterial cell to process the nascent membrane protein correctly may result in the production of aggregated material that fail to insert in the membrane and that end up in inclusion bodies^3,30^. The similar overexpression of PatA/PaB in the membrane fraction of the various strains suggested *a priori* that PatA/PatB was correctly folded in the experimental conditions tested here. However, misfolded material can also be found associated with the membrane^30^, and we sought to verify whether the transporters overexpressed from the various strains were equally functional. The transport functionality was assayed at 25 °C by using Hoechst 33342, which is a fluorescent drug translocated by PatA/PatB and many drug transporters (e.g. P-glycoprotein^31^). Transport was energized by GTP, which is the preferred energy source for PatA/PatB^14^. Strikingly, Hoechst 33342 was much better transported (>3 fold) when PatA/PatB was expressed in C41(DE3) strain as compared to the other strains (Fig. 1B,C). In the absence of PatA/PatB (see Fig. S1 for coomassie-stained gel), C41(DE3) membranes display no Hoechst transport at that temperature (Fig. 1B). Next, we induced the expression of the transporter at exponential phase (when OD_600 nm_ was between 0.3 and 1), for 5 hours at 25 °C. While expression of PatA/PatB was much less efficient in the C41(DE3) strain (Fig. 1D), transport of Hoechst 33342 was fairly similar from all three strains tested when equivalent amounts of total membrane proteins were used (Fig. 1E), showing again that the transport activity of PatA/PatB, i.e. related to the total amount of the transporter in the membrane, was far superior when expressed in C41(DE3) strain. In order to address the possibility that the rate of transport reached a saturation level in this experiment, 4 times less membrane vesicles were used but a similar level of transport activity was again found with the three different strains (Fig. S2). Thus, despite being addressed to the membrane, a large fraction of PatA/PatB is not functional in BL21(DE3) or T7 express cells.

### Detergent solubilization of PatA/PatB

**Figure 2:**
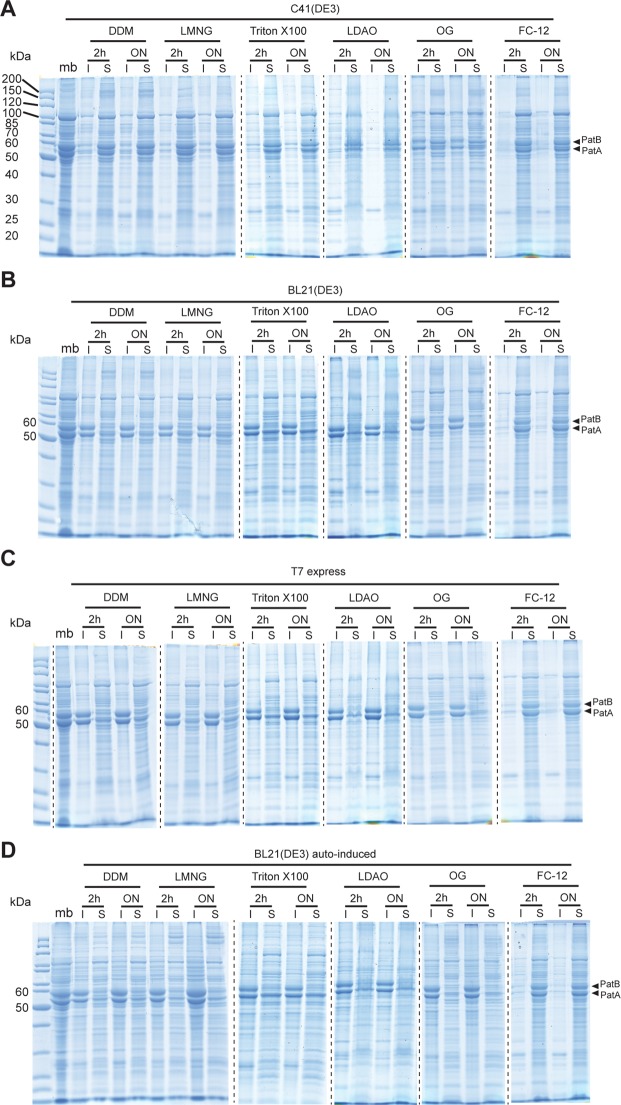
Detergent solubilization of overexpressed PatA/PatB from membranes isolated from various strains. A selection of detergents was employed to extract PatA/PatB from the membranes (10 µg of total proteins) displayed in Fig. 1A, either for 2 h or for overnight incubation. After ultracentrifugation, the soluble (S) and insoluble (I) fractions were submitted to 12% SDS-PAGE. Twenty µg of proteins were loaded in the membrane lane (mb). (**A**) PatA/PatB solubilization from C41(DE3) membranes. (**B**) PatA/PatB solubilization from BL21(DE3) membranes. (**C**) PatA/PatB solubilization from T7 express membranes. (**D**) PatA/PatB solubilization from BL21(DE3) membranes prepared after auto-induction expression. Dash lines delineate cropped images from different parts of the same gel or from different gels.

Since the conformation of PatA/PatB appears dependent on the strain used for overexpression, we next investigated the capacity of different detergents to solubilize the transporter from these three strains. Strikingly, detergents like n-dodecyl-β-D-maltoside (DDM), lauryl maltose neopentyl glycol (LMNG), triton X-100 and lauryldimethylamine N-oxide (LDAO) solubilized much better PatA/PatB overexpressed in C41(DE3) as compared to the two other strains (Fig. 2). The n-octyl-β-D-glucoside (OG) was less efficient to solubilize PatA/PatB from the C41(DE3) strain but was inefficient when used with membranes prepared from the two other strains. In contrast, the harsher detergent Fos-Choline 12 (FC12) was able to solubilize the transporter regardless of the strain used for the overexpression. These experiments suggest that PatA/PatB is less extractable by milder detergents when it is not optimally folded and functional in membranes.

### Overexpression and functionality of BmrA in several *E. coli* strains

**Figure 3:**
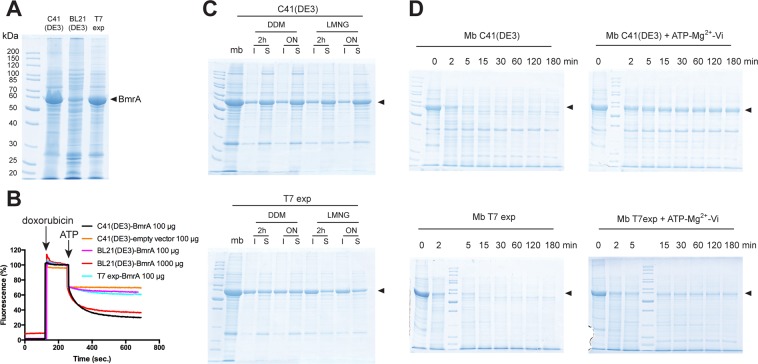
Overexpression of BmrA in the membrane of various *E. coli* strains and functionality of the transporter. The expression of the transporter was induced by IPTG in exponential phase for 4 h. (**A**) Membrane protein expressions were visualized by 12% SDS-PAGE stained with Coomassie blue. Twenty µg of proteins were loaded in each lane. (**B**) Doxorubicin transport assays with inverted membrane vesicles prepared from the various strains. For the assays, 100 µg of total proteins were used for C41(DE3) and T7 express membranes, while 100 or 1000 µg were used for BL21(DE3) membranes. A representative experiment of 2–3 replicates is shown. (**C**) Detergent solubilization of membranes containing BmrA overexpressed from various strains. DDM or LMNG detergents were employed to extract BmrA from the membranes (10 µg of total proteins), either for 2 h or for overnight incubation. After ultracentrifugation, the soluble (S) and insoluble (I) fractions were submitted to 12% SDS-PAGE. Twenty µg of proteins were loaded in the membrane lane (mb). (**D**) Limited proteolysis of BmrA by trypsin. Membranes (Mb) from C41(DE3) or T7 express strains containing overexpressed BmrA were submitted to trypsin digestion for the indicated times, in the absence of ligand or after an incubation with ATP, Mg^2+^ and orthovanadate.

The choice of overexpressing strain appeared critical for the functionality of PatA/PatB. Does this hold true for other membrane proteins whose activity can be directly and easily probed before any detergent extraction? We took advantage of functional tests developed with BmrA, a multidrug ABC transporter from *B. subtilis*^18^ that we historically expressed in C41(DE3) strain^32^, to address this question. In contrast to PatA/PatB^13,14^, BmrA is a homodimeric transporter^33^ and it is also classically powered by ATP binding and hydrolysis. BmrA was efficiently overexpressed in C41(DE3) and T7 express strains, while the level of membrane expression was lower in BL21(DE3) strain (Fig. 3A). Of note, BL21(DE3) was also difficult to transform by the plasmid carrying BmrA, with 10 to 100 times less transformants as compared to the other strains. We performed transport assays with doxorubicin, and not Hoechst 33342, for the following reason. Since BmrA is less expressed in BL21(DE3) membranes, we aimed to add a higher amount of these membranes in transport assays to have similar amounts of BmrA. We estimated by coomassie-stained SDS-PAGE that BmrA was about 10 times less expressed in BL21(DE3) strain as compared to the two other strains (Fig. S3). Hoechst fluorescence is highly dependent on the amount of membranes because its quantum yield drastically increases in hydrophobic environments^31^, whereas doxorubicin fluorescence is not sensitive to that and is simply quenched by DNA when transported inside the inverted membrane vesicle^34^. In addition, there is no basal transport of doxorubicin in *E. coli* membranes^18^, giving us the opportunity to freely increase the quantity of BL21(DE3) membranes in doxorubicin transport assay. As observed in Fig. 3B with equivalent amounts of BmrA in the assay medium (~10 times more membrane of BL21(DE3) as compared to the other strains), BmrA efficiently transports doxorubicin when expressed in C41(DE3) or BL21(DE3) strains, but not in T7 express strain. As shown previously for PatA/PatB, the DDM and LMNG detergents solubilized BmrA more efficiently when expressed in C41(DE3) strain as compared to T7 express strain (Fig. 3C), while FC12 solubilized nearly all BmrA in each strain (Fig. S4A). Of note and in contrast to PatA/PatB, Triton X100 solubilized 50% of BmrA in all strains (Fig. S4B). To check whether BmrA could properly cycle between the inward- and outward-facing conformations in membranes, limited digestion with trypsin was carried out as performed before^19^. In C41(DE3) membranes, BmrA in the apo (inward-facing) conformation was rapidly digested by trypsin, i.e. within 5 min (Fig. 3D). In the closed vanadate-trapped conformation (outward-facing), however, most of BmrA was resistant to this protease for about 180 min. This result corroborated the ability of BmrA to switch between two very different conformations when functionally overexpressed in C41(DE3) membranes^19^. In contrast, less than 30% of BmrA (quantification made using ImageJ software) expressed in T7 express membranes switched from the inward- to the outward-facing conformations as it was rapidly digested by trypsin in both conditions, apo and vanadate-trapped conformations. Of note, ∼30% of BmrA expressed in T7 express strain was solubilized by LMNG (Fig. 3C). Thus, these results suggest that the majority of the protein was improperly folded in this strain.

### Overexpression and functionality of BmrA-GFP fusions in *E. coli* strains

**Figure 4:**
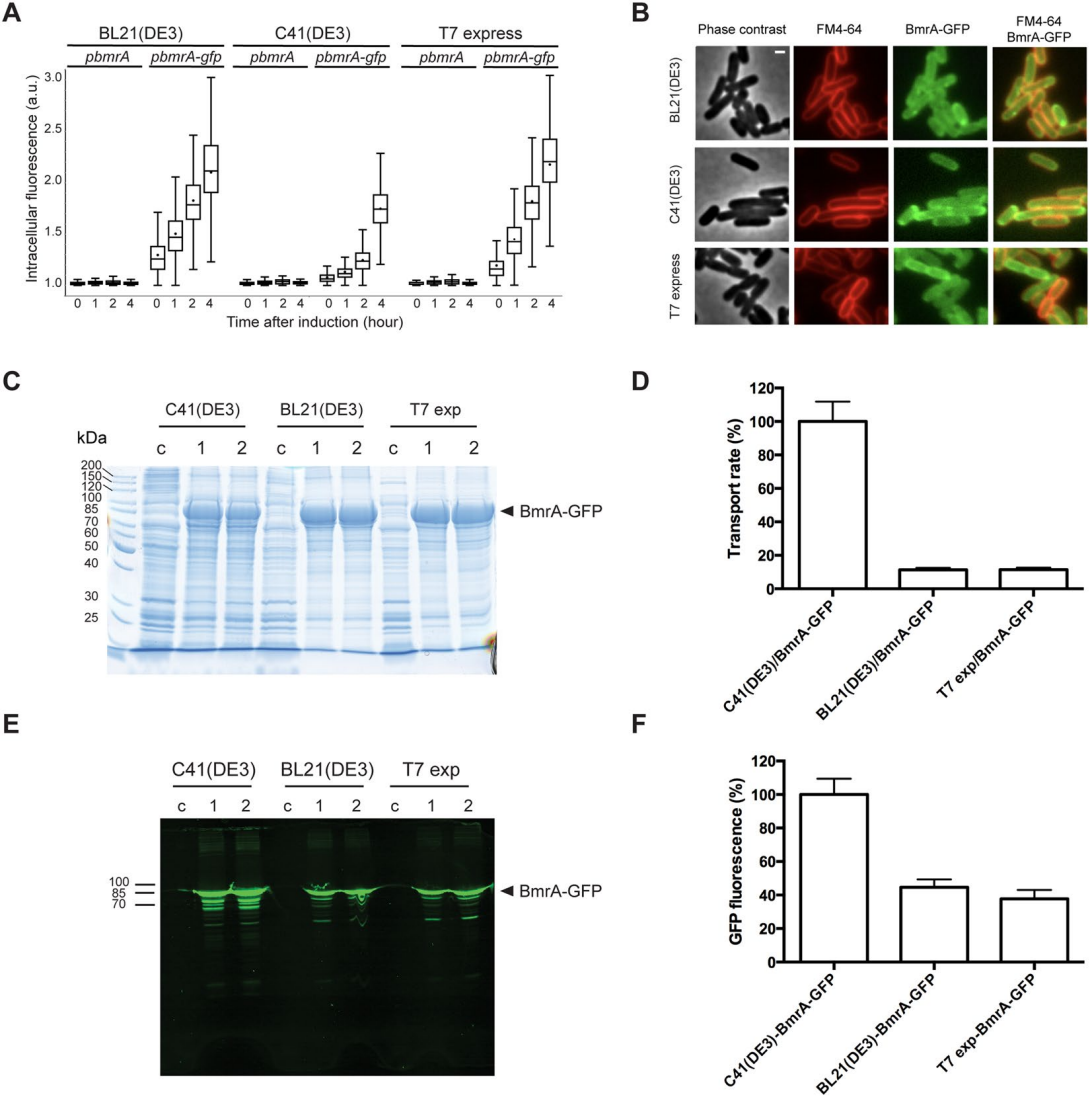
Overexpression of BmrA-GFP fusions in various *E. coli* strains and functionality of the transporter. (**A**) Analysis of BmrA-GFP production by live-cells fluorescence microscopy. Quantification of GFP intracellular signal was performed in BL21(DE3), C41(DE3) and T7 express strains containing plasmids that encode IPTG-inducible BmrA-GFP (*pbmrA-gfp*) or BmrA (*pbmrA*) proteins. Box-and-whisker plots show the statistical distribution of cellular fluorescence normalised to background before, and 1, 2 and 4 h after addition of IPTG (0.7 mM) (between 600 and 1800 cells analysed). (**B**) BmrA-GFP membrane localisation. Microscopy images taken 3 h after IPTG induction show BmrA-GFP (green channel) colocalisation with FM4-64 membrane-staining dye (red channel) in all three strains containing *pbmrA-gfp* plasmid. Scale bar, 1 μm. (**C**) membrane fractions were isolated from the strains after 4 h of induction and analyzed by 12% SDS-PAGE with Coomassie blue staining. Two preparations of BmrA-GFP (indicated as 1 and 2) and one control preparation with an empty vector (indicated as c) were analyzed for each strain. Twenty µg of proteins were loaded in each lane. (**D**) Doxorubicin transport rates with inverted membrane vesicles prepared from the various strains. Rates were calculated from initial slopes (Fig. S5). The data represent the average of triplicates, and error bars indicate the standard deviation. (**E**) In-gel fluorescence of BmrA-GFP was scanned with a typhoon imager. (**F**) Green fluorescence displayed by BmrA-GFP in membranes, as quantified with a spectrofluorometer and corrected from the amount of BmrA-GFP in each membrane (as determined in Figs S6 and S7). The data represent the average of triplicates, and error bars indicate the standard deviation. Panels 4C–F were analyzed with the same batches of membrane preparation.

Fusing GFP to the C-terminus of membrane proteins has been widely used as a strategy to monitor the levels of membrane proteins properly addressed to the membrane^3^. The GFP does not fold properly and does not fluoresce when the overexpressed protein ends up in inclusion bodies^12^. Although this tool does not provide direct information on the functionality of the synthesized proteins, GFP fluorescence was correlated with the functional overexpression of a number of fused membrane proteins^30^. We thus sought to determine if this assumption was correct with BmrA as a case study. Previously, The functionality of BmrA fused to GFP was shown to be retained in *B. subtilis*^35^. First, overexpression of a BmrA-GFP fusion was monitored as a function of induction time in C41(DE3), BL21(DE3) and T7 express strains (Fig. 4A). By looking at the kinetics, the induction of BmrA-GFP was slower in C41(DE3) as compared to the other strains in which fluorescence raised much earlier. Moreover, after 4 h of induction the level of fluorescence was very high in both the T7 express and the BL21(DE3) strains, but a bit reduced in the C41(DE3) strain. In these three strains, the BmrA-GFP fusion appears to be present in, or close to, the membrane (Fig. 4B), suggesting that a significant fraction of the fusion protein was both properly targeted to the membrane and functional. Next, the amount of BmrA-GFP fusion in the membranes of the different strains was assessed. It was found to be fairly similar for the three strains, although a bit reduced for C41(DE3) strain (Fig. 4C). Nevertheless, as shown previously for BmrA alone, the fusion BmrA-GFP was ~10 times more efficient at transporting doxorubicin when expressed in C41(DE3) strain (Figs 4D and S5). We also analyzed the levels of GFP fluorescence in each membrane sample after migration on a SDS-PAGE, since it was reported that correctly folded GFP is not denatured in SDS-polyacrylamide gel under certain conditions^30^. We observed a higher fluorescence level in the C41(DE3) membranes as compared to the other strains (Fig. 4E). Furthermore, we measured the fluorescence associated with the non-denatured membranes with a spectrofluorometer (Fig. S6). After subtracting the background fluorescence of the control membranes, the latter quantification allowed us to determine the GFP fluorescence associated with a normalized amount of BmrA from each membrane source (Figs S7 and 4F). Despite the fact that the transport activity of BmrA-GFP is reduced by more than 90% when expressed in BL21(DE3) and T7 express strains (Fig. 4D), the fused BmrA-GFP fluoresces to about 40% when expressed in T7 and BL21(DE3) strains as compared to C41(DE3) strain (Fig. 4F). We found similar results (∼35%) by quantifying the in-gel fluorescence of the full-length BmrA-GFP by typhoon fluorescence imaging and ImageJ. Therefore, while a higher GFP fluorescence intensity was visible in the C41(DE3) strain (Fig. 4E) thereby correlating with a higher functionality of BmrA in this strain, a substantial amount of GFP fluorescence was visible in the membrane of the strains in which the functionality of BmrA was severely impaired. Therefore, these data suggest that fluorescence and thus folding of GFP can occur independently of the folding of the target membrane protein fused to its C-terminus.

### Expression in Lemo21(DE3) strain

**Figure 5:**
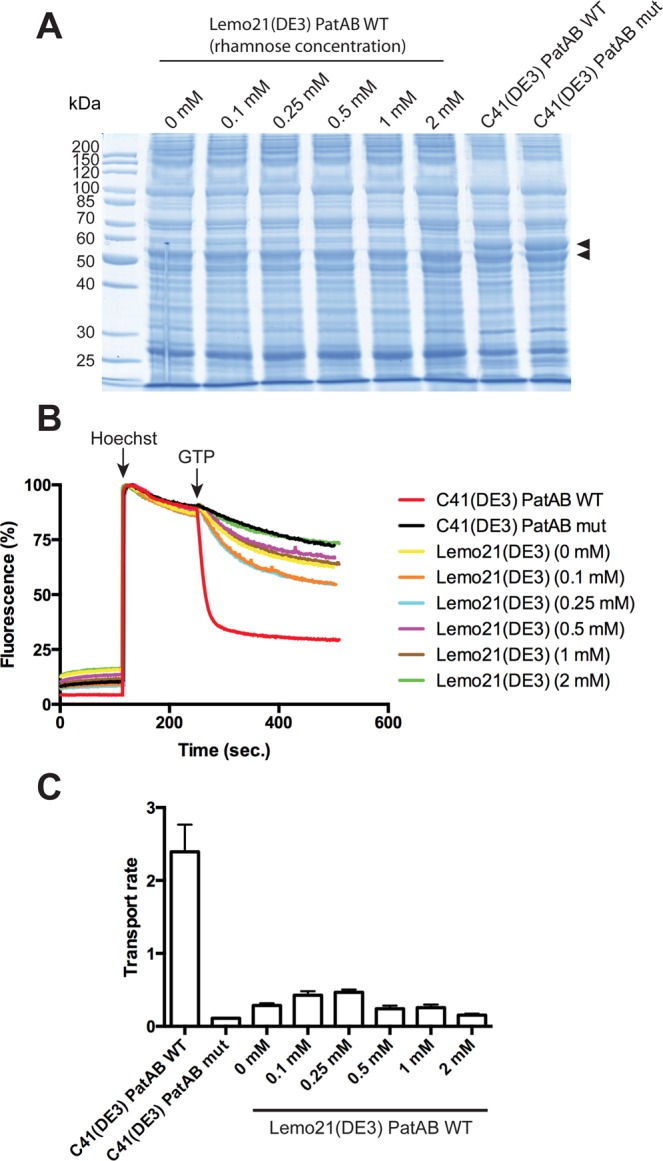
Expression of PatA/PatB in Lemo21(DE3) strain and functionality of the transporter. (**A**) Membrane proteins in Lemo21(DE3) and C41(DE3) strains were visualized by 12% SDS-PAGE stained with Coomassie blue. Twenty µg of proteins were loaded in each lane. As a negative control, a mutant of PatA/PatB was used in which the conserved Walker-A lysine was mutated in each subunit (PatA-K367A/PatB-K388A). (**B**) Hoechst 33342 transport assays with inverted membrane vesicles prepared from the various strains. 50 µg of total membrane proteins were used for each assay. (**C**) Hoechst 33342 transport rates as measured by initial slopes following GTP addition and calculated from panel B. The data represent the average of triplicates, and error bars indicate the standard deviation.

This strain was engineered to provide an “All in One” platform for membrane protein overexpression, since the activity of the T7 polymerase can be controlled by rhamnose concentration^10^. Surprisingly, the expression of PatA/PatB was much lower in Lemo21(DE3) as compared to C41(DE3) strain (Fig. 5A), although the IPTG induction was performed in a similar way (induction at OD ~1.7 during overnight at 25 °C in TB media). Accordingly, drug transport was much faster in the latter strain (Fig. 5B), although membranes prepared from Lemo21(DE3) cells displayed a bell-shape rate of transport dependent on the rhamnose concentration used during induction (Fig. 5C). We performed a western-blot analysis, which suggests that the highest transport seen at 0.1–0.25 mM rhamnose corresponded to a higher expression of PatA/PatB in these conditions (Fig. S8). Moreover, ~5 times less C41 membranes was sufficient to detect a similar band intensity, suggesting that PatA/PatB was about 5 times more expressed in the membranes of C41(DE3) strain. Knowing that drug transport was approximately 5 times faster in C41(DE3) membranes (Fig. 5C), these results suggest a similar quality of PatA/PatB expressed in C41(DE3) and Lemo21(DE3) strains, although the level of production was much higher in the former. The expression of BmrA was also attempted in Lemo21(DE3) cells with various rhamnose concentrations, in TB medium (25 °C and 37 °C) or LB medium (37 °C). At 25 °C in TB, none of the rhamnose conditions permitted a high overexpression of the transporter, in contrast to the C41(DE3) strain (Fig. S9A). However, while transport activity from C41(DE3) membranes was ~3 times higher as compared to the highest one from Lemo21(DE3) membranes, protein quantification by Western blot showed that BmrA was markedly more abundant in the C41(DE3) as compared to the Lemo21(DE3) strain, much more than 3 fold in the former strain. This suggested that the lower expression in Lemo21(DE3) strain at 0.5 mM rhamnose produced a higher fraction of functional transporters. When the induction was performed at 37 °C in TB (Fig. S9B), we found some conditions in which Lemo21(DE3) strain overexpressed BmrA as well as in C41(DE3) strain (or better, ~1.5 fold at 0.5 mM rhamnose, see Western Blot), and the Lemo21(DE3) membranes were about 3 times more active. When the induction was performed at 37 °C in LB (Fig. S9C), the overexpression of BmrA in Lemo21(DE3) strain showed a bell-shape that correlated with transport activities. At 0.2 and 0.5 mM rhamnose, both the expression and transport of BmrA in the Lemo21(DE3) strain was ~3 times higher than in C41(DE3) strain.

### Functionality of the transporters purified from various strains

**Figure 6:**
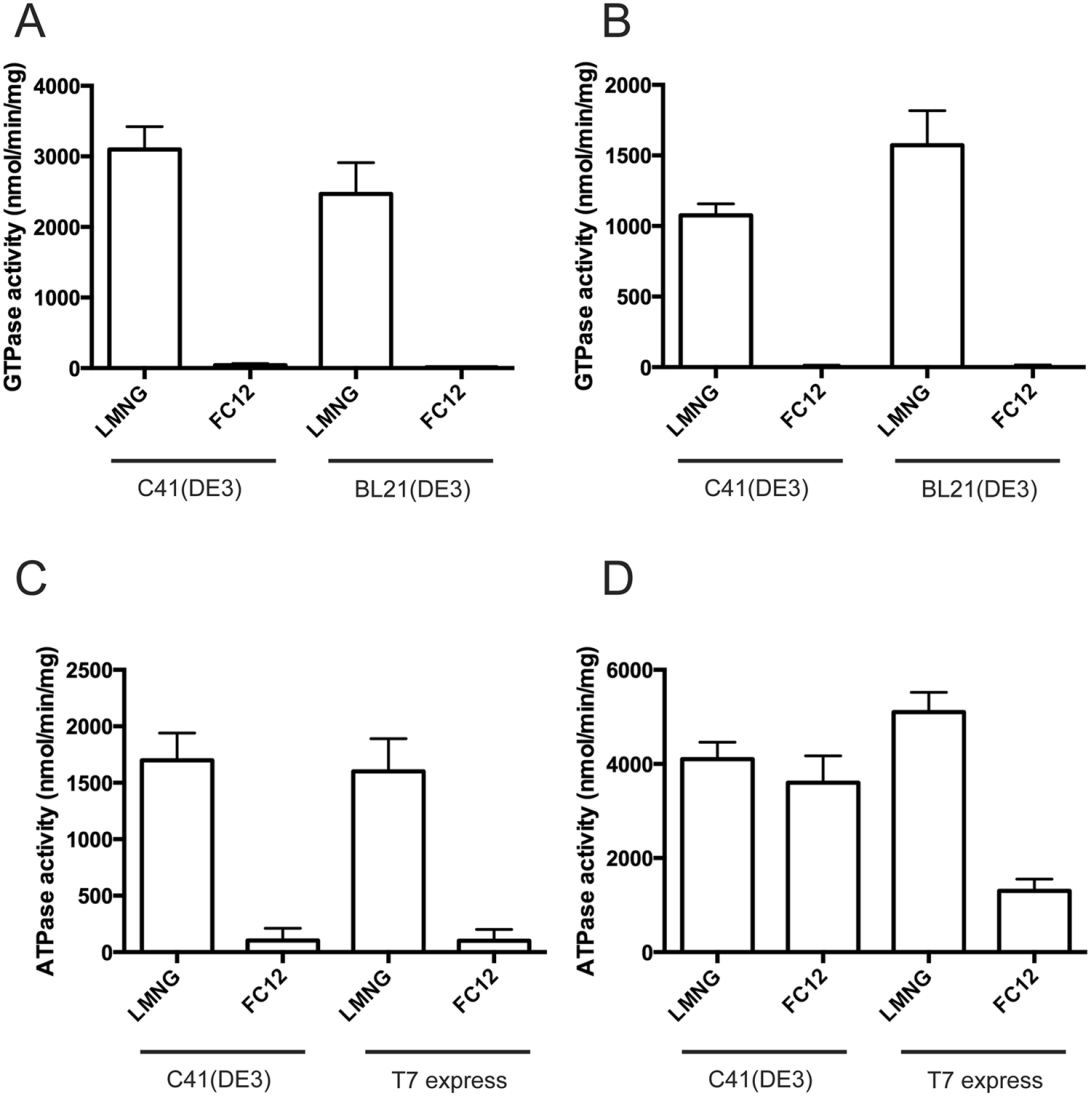
NTPase activities of purified PatA/PatB and BmrA in detergent or after reconstitution into proteoliposomes. (**A**) GTPase activities of PatA/PatB solubilized from C41(DE3) or BL21(DE3) with the indicated detergent and purified by Ni-affinity. (**B**) GTPase activities of PatA/PatB solubilized from C41(DE3) or BL21(DE3) with the indicated detergent, purified and reconstituted into proteoliposomes. (**C**) ATPase activities of purified BmrA solubilized from C41(DE3) or T7 express with the indicated detergent and purified by Ni-affinity. (**D**) ATPase activities of BmrA solubilized from C41(DE3) or T7 express with the indicated detergent, purified and reconstituted into proteoliposomes. The data represent the average of triplicates (panels A and B) or six replicates (panels C and D), and error bars indicate the standard deviation.

As described above, the functionality of PatA/PatB and BmrA is highly influenced by the choice of the T7 expression strain. Because milder detergents seem to extract a higher proportion of active transporters in the host membrane, this raises two important questions. First, could a mild detergent indeed selectively extract the population of functional membrane proteins? Second, since a detergent like FC12 was able to extract the transporters irrespective of the quality of the transporter, how does such detergent preserve or affect the functionality of the transporters? To address these questions, we solubilized and purified PatA/PatB that was expressed in C41(DE3) and BL21(DE3) strains, with either LMNG or FC12 (see Table S1 for purification yields). The first noticeable observation we made was that, in contrast to LMNG that seems to preserve a constant stoichiometry of the PatA and PatB subunits, FC12 at least partially dissociated PatA and PatB during the elution of the affinity chromatography leading to an uneven distribution of the two subunits in the different fractions (Fig. S10). We next characterized the GTPase activity of the purified transporters, both in detergent or reconstituted in proteoliposomes. Interestingly, the PatA/PatB population solubilized and purified in LMNG from both strains remained highly active (Fig. 6A). Upon reconstitution, although the GTPase activities were reduced by 2–3 fold, as reported before^14^, they remained similar for PatA/PatB overexpressed and purified from both strains (Fig. 6B). In contrast, no significant GTPase activity could be measured when FC12 was used to purify PatA/PatB, regardless of the strain used, and even after reconstitution into proteoliposomes (Fig. 6A,B). For BmrA (see Table S1 for purification yields), the activity of the protein purified from each strain with LMNG was also similar (Fig. 6C), even after reconstitution (Fig. 6D), except that 2–3 fold higher ATPase activities were measured upon proteoliposome reconstitution. When FC12 was used to solubilize and purify BmrA from either strain, a much-reduced ATPase activity was observed as compared to LMNG (~7 times) (Fig. 6C). BmrA purified by FC12 from the C41(DE3) regained a full ATPase activity upon reconstitution, comparable to that obtained with LMNG (Fig. 6D). In contrast, BmrA extracted by FC12 from the T7 express did not regain a level of ATPase activity similar to that obtained from the LMNG treated sample upon reconstitution. The ATPase activity of the FC12 treated BmrA was ~2–3 times lower than the LMNG treated BmrA. Presumably, this is due to the fact that FC12 extracted both folded and unfolded forms of BmrA from the T7 express cells. Upon reconstitution in proteoliposomes, the inactive co-purified fraction failed to regain activity. On the other hand, in the C41(DE3), a large majority of BmrA is properly folded and can be solubilized using either LMNG or FC12. Although the latter strongly inhibits the ATPase activity of BmrA, removal of the detergent during the reconstitution process allowed BmrA to recover its activity.

## Discussion

Overexpression represents the first major bottleneck in structural and functional studies of integral membrane proteins. In the recent years, substantial progress has been accomplished in producing both prokaryotic and eukaryotic membrane proteins in bacterial hosts, especially *E. coli* but also *Lactococcus lactis* and *B. subtilis*^3^. Notably, it was shown that many strains with strongly improved membrane protein overexpression ability could be selected for^9,36–42^ or engineered^43–47^. However, the tools to assess the overall quality of the overexpressed proteins are limited. The most commonly used strategy has been to fuse GFP to the C-terminal end of membrane proteins^12,48^, although it should be kept in mind that fusing fluorescent protein tags may introduce artifacts in the localization and/or stability of the proteins of interest^49^. It was reported that proper folding and fluorescence of GFP fused to the C-terminus of a target protein depends on the appropriate folding of the latter, and thus only folded fusion protein is fluorescent. Instead, if the fusion protein is present in inclusion bodies, the GFP does not fold properly and thus does not fluoresce. Methods employing GFP fusion seem therefore extremely useful to assess a first quality control for membrane protein production^30,36^. Membrane protein biogenesis is indeed complex and tightly controlled in order for the synthesized membrane proteins to be correctly targeted, inserted and then folded into the membrane^50,51^. GFP-fusions also allowed using minimal amounts of detergent-solubilized whole cells/membranes to rapidly assess both membrane protein production levels and their degree of monodispersity using fluorescence-detection size-exclusion chromatography precrystallization screening^52^. However, these methods do not directly provide any information on the functionality of the proteins that are overexpressed. Here, we took advantage of two different membrane proteins for which functional assays can be easily and directly performed at the level of membrane vesicles, i.e. before their solubilisation with detergents that might compromise their active conformation. Our study clearly illustrates that membrane proteins that are seemingly properly addressed to the membrane is not a guarantee for quality. In addition, when working with the widely employed GFP fusion strategy, we showed a lack of correlation between the GFP fluorescence and the functionality of BmrA, revealing that the two proteins in the chimeric construct can fold independently of each other. At the cell level (Fig. 4A), BL21(DE3) and T7 express displayed higher fluorescence intensity than C41(DE3), which would encourage investigators toward choosing the former strains for downstream work. When the membrane fraction was isolated (Fig. 4E), a higher GFP fluorescence was associated with C41(DE3) membranes, although we demonstrated that about 35–40% of fluorescence was associated with inactive BmrA in the other strains (Fig. 4D,F). To explain this apparent discrepancy between cell and membrane levels, it is possible that some fluorescent BmrA-GFP was not targeted to the membrane in BL21(DE3) and T7 express cells and/or that a fraction of fluorescent BmrA-GFP in their membranes was very unstable and degraded during membrane preparation. Regardless of the explanation for this difference, it is clear that considering just the global level of GFP fluorescence for fusion of membrane proteins could be quite misleading. It is very likely that this downside of using GFP fusion might also be a problem when one works with soluble proteins.

Overall, we found that PatA/PatB and BmrA transporters appear much more functional when expressed in the C41(DE3) strain as compared to the BL21(DE3) and T7 express strains, as evidence by a series of assays. First, real-time drug transport assays clearly showed that these transporters are more active in the membranes prepared from this strain. Second, limited proteolysis on BmrA suggested its ability to operate conformational changes when expressed as an active form in membranes, but not as inactive ones. Third, a screening with a variety of detergents indicated that functional membrane proteins could be solubilized from the membrane by milder detergents, whereas inactive proteins are generally less extractable by these detergents. The LMNG and DDM detergents were indeed able to extract high amounts of the transporters from the membranes of the best strains, while little amounts of transporters were solubilized from the strains with a poorer capacity for functional overexpression. However, the extracted fraction of proteins was active in all cases. In contrast, a harsh detergent such as FC12 was able to extract the protein irrespective of the membrane strain. Neither PatA/PatB nor BmrA was active in FC12, although the activity of the latter could be almost fully recovered upon reconstitution in proteoliposomes. These observations are notable because direct functional tests are not always available for overexpressed membrane proteins, and this simple detergent assay may provide a valuable tool to suggest whether heterologously expressed membrane proteins are indeed properly folded. Interestingly, similar observations were recently described for membrane proteins expressed in eukaryotic systems^53^. The proper choice of detergent is fundamental for functionality of membrane proteins and protein structure determination^21,54,55^. The right balance between the ability of the detergent to solubilize the protein of interest and stabilize its native structure is key for such goals^55^. Indeed, detergents can compete with stabilizing intramolecular interactions within membrane proteins, leading to their inactivation^56–58^ or the alteration of their properties that can be recovered once reconstituted in lipids^59,60^. Although Foscholine detergents appear to be detrimental for the native conformation of many membrane proteins^61,62^, there are a few examples in which the functionality of the purified proteins could be regained after incorporation into proteoliposomes^54^, as found here for BmrA. This is in agreement with a previous study where the ATPase activity of BmrA solubilized in FC12 could be restored after detergent exchange^63^. In contrast, the heterodimeric transporter PatA/PatB appears much more sensitive to FC12, as also reported for a related heterodimeric transporter from *B. subtilis*, BmrCD^64^. As seen with our study, the choice of the overexpressing strain can also influence the choice of detergent for further applications, since milder detergents can both extract and maintain the protein function when used with the proper expression strain.

While BL21(DE3) strain was originally developed for the production of soluble proteins, overexpression of proteins, and especially membrane proteins, may have toxic effects and induces a wide range of physiological perturbations^6,10,65^. In 1996, Miroux and Walker cleverly isolated mutant derivatives of BL21(DE3) with improved yields and reduced toxicity for membrane protein production^9^. These C41(DE3) and C43(DE3) strains are now widely used to produce membrane proteins^66^, although they do not improve yields for all membrane proteins. Very recently, Miroux’s laboratory selected additional BL21(DE3) mutants, C44(DE3) and C45(DE3) strains, that appeared better suited to overproduce some eukaryotic transporter^36^. Regarding the C41(DE3) and C43(DE3), it was much later shown that mutations in the lacUV5 promoter governing expression of the T7 RNA polymerase were key to the improved membrane protein production characteristics of these strains, because such mutations would result in the production of much lower amounts of T7 RNA polymerase upon induction than in BL21(DE3) strain^10^. Based on this observation, a derivative strain of BL21(DE3), termed Lemo21(DE3), was constructed in which the activity of the T7 RNA polymerase can be precisely tuned by its natural inhibitor T7 lysozyme. In Lemo21(DE3) the gene encoding the T7 lysozyme is on a plasmid under the control of a well titratable rhamnose promoter, thereby offering the possibility of a wide window of expression levels^10,11^. It has been proposed that subsequent lower synthesis rates of the mRNA for the target membrane protein ensure that the capacity of the Sec-translocon machinery is sufficient to incorporate the produced proteins in the membrane, thereby decreasing toxicity^8,10,11,67^. Although this hypothesis is certainly true, it may not be the sole explanation. Our results show that the amounts of PatA/PatB and BmrA incorporated in BL21(DE3) and T7 express membranes can be as high (or even higher) as in C41(DE3) membranes, apparently suggesting that the Sec-translocon could handle in all strains the insertion of these overexpressed membrane proteins. However, more PatA/PatB and BmrA proteins are appropriately folded in C41(DE3) strain than in the other strains. How can we explain this? First, membrane protein insertion in the membrane can occur without translocon as seen with cell-free translation systems supplemented with liposomes or vesicles, although the mechanism is poorly understood^68–70^. It is thus possible that, under the non-physiological high level of protein synthesis (as here with the T7 system), some membrane proteins may not be processed by the Sec machinery and insert/fold improperly into the membrane. Second, transcription and translation are normally coupled in bacteria^71^. The *E. coli* RNA polymerase (RNAP) transcribes approximatively 60 nucleotides per second, and a ribosome initiating translation on the mRNA nascent chain proceeds with a rate of 20 amino acids per second, i.e. 20 codons (60 nucleotides) per second. Therefore, during active transcription/translation, there is no significant gap between the transcriptase and the following ribosome. The nascent mRNA chain cannot form secondary structures that would prevent translation elongation or transcription via R-loop formation. Moreover, the presence of ribosomes also protects the mRNA against RNAseE degradation^72^. However, the T7 RNAP is up to eight times faster than *E. coli* RNAP, thus breaking the tight coupling between transcription and translation with possible adverse consequences^5^. Strong secondary RNA structures can form and hinder the path of the translating ribosome along the mRNA, likely impairing the co-translational folding and thus the yield of active proteins^73^. As shown in recent years, translation pace and its changes can regulate protein folding, and native translation is in fact uneven along each mRNA^74^. Most likely, a combination of all these factors is responsible for the impaired functionality of membrane proteins when overexpressed from high levels of transcription. The fine-tuning of transcription, mRNA stability and translation is often required for optimal protein synthesis^73,75,76^. Although our study only focused here on two membrane proteins, our data suggested that the Lemo21(DE3) strain did not overexpress well at 25 °C, as compared to the C41(DE3) strain. However, we found some conditions in which the Lemo21(DE3) strain outperform the C41(DE3) strain for functional overexpression of BmrA at 37 °C. Overall, our study clearly advises against choosing a bacterial strain based solely on expression levels in the membranes, even though some harsh detergents are able to extract these proteins. Although activity assays are the gold standard to assess quality, alternate tools can be used when functional tests are not readily achievable. Limited proteolysis can assess conformational changes, and detergent solubilization can provide valuable hints regarding the quality of overexpressed membrane proteins. With the technical advances and successes of structural methods such as X-ray crystallography, Cryo-Electron Microscopy and solid-state NMR, the high-resolution structures of membrane proteins is at much closer reach than before, but the overexpression in a functional and homogeneous state remain the second hurdle encountered for the structural analysis and biochemical characterization of membrane proteins. Identifying and counteracting the pitfalls of membrane protein expression remain at stake and will require the efforts from all the community with a broad range of case studies.

## Methods

### Bacteria and growth conditions

C41(DE3), BL21(DE3) and T7express strains were transformed either by pETDuet-1-PatA/PatB^13^ or pET23b-BmrA^32^. For PatA/PatB, a fresh colony was inoculated into 500 mL of Terrific broth medium containing 100 µg/ml of ampicillin and grown at 37 °C with shaking at 190 rpm. Protein expression was induced by 0.7 mM of IPTG either when the OD_600 nm_ reached 0.3–1 or 1.6–2, and during respectively 5 h or overnight at 25 °C with shaking at 190 rpm. For auto-induced BL21(DE3), a pre-culture was done in the morning from an individual clone inoculated into 100 mL of LB at 37 °C with shaking at 200 rpm. In the evening, the preculture was inoculated into 500 mL of auto-induced medium (1% N-Z-amine, 0.5% yeast extract, 25 mM Na_2_HPO_4_, 25 mM KH_2_PO_4_, 50 mM NH_4_Cl, 5 mM Na_2_SO_4_, 2 mM MgSO_4_, 0.05% Glucose, 0.2% Lactose, 0.5% Glycerol) such as the initial OD_600 nm_ was about 0.2. The culture was left overnight at 25 °C with shaking at 190 rpm. For BmrA, a freshly transformed colony was inoculated into 1 L of Terrific broth containing 100 µg/ml of ampicillin. The flask was then incubated at 25 °C and 180 rpm overnight. The next morning, when the OD_600 nm_ reached 0.6, overexpression was induced by addition of 0.7 mM IPTG (final concentration) and the culture was induced for 4 h at 25 °C and 180 rpm. Then, the medium was centrifuged at 4000 × g for 20 min at 4 °C to obtain the pellet of bacteria which was either frozen at −80 °C or used directly.

### Membrane Preparation

Bacteria collected by low speed centrifugation were resuspended in a buffer (50 mM Tris-HCl pH 8.0, 5 mM MgCl_2_, 1 mM dithiotreitol, protease inhibitor cocktail tablet (Roche)). Bacteria were then lysed by three successive passages through a Microfluidizer^TM^ at 18 000 psi. Unbroken cells were removed by a 30 min centrifugation at 15,000 × g. Membrane vesicles were collected by an ultracentrifugation at 150,000 × g during 1 h. Pellets were re-suspended in a buffer (50 mM Tris-HCl pH 8.0, 1.5 mM EDTA, 1 mM dithiotreitol) and centrifuged again 1 h at the same speed. Final membrane vesicles were resuspended in a buffer (20 mM Tris-HCl pH 8.0, 1 mM EDTA and 300 mM sucrose) then flash-frozen in liquid nitrogen and conserved at −80 °C. Protein concentrations in membrane vesicles were measured by a BCA assay and protein overexpression was checked by SDS-PAGE subsequently stained by Coomassie Brilliant Blue.

### Solubilization tests

Detergent solubilization was performed in a final volume of 200 µL at 2 mg/mL of membrane protein in a buffer (50 mM Tris-HCl pH 8.0, 100 mM NaCl, 15% Glycerol) at 4 °C. Various detergents were used (LMNG, DDM, Triton X100, LDAO, OG, Fos-Choline-12) at different concentrations (1% for all of them, except for OG where 2% was used). The solubilization was performed either during 2 h or overnight. Solubilized proteins were collected in the supernatant after an ultracentrifugation at 150,000 × g during 1 h. The insoluble pellets were resuspended in equal volume of the same buffer. 5 µL of samples in Laemmli buffer were loaded and migrated in a 12% SDS-PAGE during 1 h at 200 Volts and proteins were stained by Coomassie Brilliant Blue.

### Transport assays

Experiments were performed in a 2 mL quartz cuvette with a final volume of 1 mL in a buffer (50 mM Hepes-KOH pH 8.0, 8.5 mM NaCl, 4 mM phosphoenolpyruvate, 60 µg/mL pyruvate kinase (Roche), 2 mM MgCl_2_) and monitored by a Photon Technology International Quanta Master I fluorimeter. Excitation and emission wavelengths were set at 355 and 457 nm, respectively, for Hoechst 33342 [2-(4-ethoxyphenyl)-5-(4-methyl-1-piperazinyl)-2,5-bis-1H-benzimidazole] or at 480 and 590 nm, respectively, for doxorubicin. After incubation at 25 °C during 1 min, inside-out membrane vesicles (IMVs) were added and the fluorescence was recorded. After 2 min, 1 µM of Hoechst 33342 or 10 µM of Doxorubicin was added. Two min later, the transport was induced by adding 2 mM of ATP or GTP and recorded during around 8–10 min until stabilization. Doxorubicin fluorescence is quenched by *E. coli* native DNA that was trapped inside the vesicles during cell disruption^18^.

### Limited proteolysis of BmrA

Inside-out membrane vesicles (IMVs) containing overexpressed BmrA were added into a buffer (20 mM Tris-HCl pH 8, 1 mM EDTA) and after 15 min of incubation, trypsin (1 µg/250 µg of BmrA) was added. For the Vi-inhibited form, before the addition of IMVs containing overexpressed BmrA, 3 mM MgCl_2_, 2 mM ATP and 1 mM Vi were also added into the buffer. Samples of 10 µl (20 µg) each were withdrawn at 0, 2, 5, 15, 30, 60, 120 and 180 min. 2.5 µl of TFA 5% was added immediately to each sample to stop the reaction. 3 µl of Laemmli buffer (5x concentrated) was then added and the samples were placed on ice before resolving them on SDS-PAGE.

### BmrA-GFP fusion

The *egfp* gene containing the mutations F64L and S65T^77^ was amplified by PCR and cloned between restriction sites SalI and NotI in the plasmid pET23b-*bmrA* thereby fusing the eGFP at the *C*-terminus of BmrA with a linker VDAAAAVDAAAA. A freshly transformed colony was inoculated into 500 mL of Terrific broth medium containing 100 µg/ml of ampicillin and grown overnight at 22 °C. Protein expression was induced by addition of 0.7 mM of IPTG when the OD_600 nm_ reached 0.6 and was pursued for 4 h at 25 °C with shaking at 180 rpm. Membranes were prepared as described above for BmrA. For in-gel analysis of GFP fluorescence, samples were prepared in a Laemmli buffer containing 0.4% SDS instead of 2%. SDS-PAGE was then run at 4 °C. After migration, gels were scanned for fluorescence with a typhoon imager. For quantification of BmrA-eGFP fluorescence in membranes, *E. coli* membranes containing 100 μg of total proteins were diluted in 1 mL of 50 mM Hepes-KOH pH 8, 8.5 mM NaCl and placed in a PTI spectrofluorometer. Samples were excited at 488 nm and their fluorescence was recorded between 500 and 600 nm. Integrated fluorescence intensities (between 500 and 530 nm) from the membranes overexpressing BmrA-GFP were corrected from the background fluorescence displayed by the control membranes prepared with empty pET23 vector (Fig. S6). A second correction was then made by estimating the amounts of BmrA overexpressed in each membrane preparation (Fig. S7) to assess the GFP fluorescence associated with a normalized amount of BmrA in membranes (Fig. 4F).

### Microscopy imaging and analysis

Several colonies were diluted into 5 mL of Terrific broth media supplemented with 100 µg/mL of ampicillin. These culture were grown overnight at 23 °C (130 rpm) such that the OD_600 nm_ on next morning was around 0.6 for all the strains. IPTG (0.7 mM final concentration) was added to the cell cultures and, after 1, 2 or 4 hours, 5 μl cell samples were transferred to a slide mounted with 1% agarose in Terrific medium^78^. When needed, cell membranes were visualized using 1 μg/mL FM4-64 (Life Technologies). Conventional wide-field fluorescence microscopy imaging was carried out on an Eclipse Ti-E microscope (Nikon), equipped with x100/1.45 oil Plan Apo Lambda phase objective, FLash4 V2 CMOS camera (Hamamatsu), and using NIS software for image acquisition. Acquisition settings were 10 ms for GFP and 10 ms for FM4-64, using 50% power of a Fluo LED Spectra X light source at 488 nm and 560 nm excitation wavelengths, respectively. Intracellular fluorescence intensity was analysed using MicrobeJ^79^ and ImageJ software (http://rsbweb.nih.gov/ij/).

### Protein expression in Lemo21(DE3) strain

Commercially competent cells (New England Biolabs) were transformed with pETDuet-1/*patA-patB*^13^ or pET23/*bmrA*^32^ and plated onto LB-agar plates containing 30 µg/mL of chloramphenicol and 100 µg/mL of ampicilline. For PatA/PatB expression, one colony was first diluted into 600 μl of Terrific Broth. Then, 100 μl of this culture was inoculated into 500 mL of Terrific Broth containing 30 µg/mL of chloramphenicol, 100 µg/mL of ampicilline and 0 to 2 mM of rhamnose. Cells were grown at 37 °C with shaking. When the OD_600 nm_ reached ~2, protein expression was induced by 0.7 mM IPTG overnight at 25 °C. Membranes were prepared as described above. For BmrA expression, an overnight LB preculture containing 30 µg/mL of chloramphenicol and 100 µg/mL of ampicilline was diluted at OD_600 nm_ = 0.1, cells were grown at 37 °C with shaking in TB or LB medium containing 30 µg/mL of chloramphenicol, 100 µg/mL of ampicilline and 0 to 2 mM of rhamnose. At OD_600 nm_ ~0.6, protein expression was induced with 0.7 mM IPTG for 4 h at 25 °C or 37 °C.

### Western Blot analysis

For PatA/PatB expression, a 12% SDS-PAGE was performed with 0.5 µg purified PatA/PatB, 30 µg of total proteins for Lemo21(DE3) membranes and 6 µg of total proteins for C41(DE3) membranes. For BmrA expression, a 12% SDS-PAGE was performed with 0.2 µg purified BmrA, 0.1 to 1 µg of total membrane proteins for Lemo21(DE3) and 0.3 µg of total membrane proteins for C41(DE3) (see legend of Fig. S9 for details). Proteins were then transferred at 300 mA for 1.5 h onto a PVDF membrane preactivated in ethanol in a buffer containing 25 mM Tris base, 192 mM glycine, 20% ethanol and 0.02% SDS. The PVDF membrane was first rinced with TBS (50 mM Tris-HCl pH 7.6, 150 mM NaCl), then blocked 1 h in buffer TBS supplemented with 0.1% tween and 10% semi-skimmed milk (powder). Next, the membrane was incubated during 1 h with a HRP-conjugated anti-pentaHis antibody (Qiagen) diluted 2000 or 10000 times (for PatA/PatB and BmrA, respectively) in a new blocking buffer. The membrane was washed 3 times with TBS buffer supplemented with 0.1% tween. Finally, the membrane was revealed with Luminata^TM^ Forte reagents from Millipore.

### Purification of PatA/PatB and BmrA

For PatA/PatB, 50 mg of total membrane proteins were solubilized in a buffer containing 50 mM Tris-HCl pH 8, 100 mM NaCl, 15% glycerol and 1% of detergent (LMNG or FC-12). After an ultracentrifugation at 150,000 × g for 1 h, solubilized proteins were applied to a 1 mL Histrap HP column (GE Healthcare) that was pre-equilibrated with a buffer containing 50 mM Tris-HCl pH 8, 100 mM NaCl, 15% glycerol and 0.02% LMNG or 0.3% FC-12. The column was then washed with the same buffer. Elution was performed with the same buffer containing an imidazole gradient up to 250 mM. Purified proteins were dialyzed in a buffer 50 mM Tris-HCl pH 8, 100 mM NaCl, 15% glycerol and 0.02% LMNG or 0.15% FC-12. The concentration of the purified PatA/PatB proteins was measured with Bradford assay. For BmrA, total membrane proteins (30 mg from C41(DE3), or 9–12 mg from T7 express) at 2 mg/mL final concentration were solubilized for 1 h in a buffer containing 50 mM Tris-HCl pH 8, 100 mM NaCl, 10% glycerol, 1 mM DTT, protease inhibitor cocktail tablet (Roche) (1 tab for 50 mL buffer) and 1% of detergent (LMNG or FC-12). After an ultracentrifugation at 150,000 × g for 1 h, solubilized proteins were applied to a 1 mL Histrap HP column (GE Healthcare) that was pre-equilibrated with a buffer containing 50 mM Tris-HCl pH 8, 100 mM NaCl, 10% glycerol, 20 mM imidazole and 0.01% LMNG or 0.3% FC-12. The column was then washed with the same buffer. Elution was performed with the same buffer containing an imidazole gradient up to 500 mM. Purified proteins were dialyzed in a buffer 50 mM Hepes-KOH pH 8, 50 mM NaCl, 10% glycerol and 0.01% LMNG or 0.15% FC-12. The concentration of the purified BmrA was measured with Nanodrop (UV absorbance at 280 nm).

### Membrane protein reconstitution

BmrA and PatA/PatB proteoliposomes were prepared as described before^20^.

### ATPase and GTPase activities

Activities were measured in 700 µL total volume in buffer Hepes-KOH 50 mM pH 8, 10 mM MgCl_2_, 4 mM phosphoenolpyruvate, 0.43 mM NADH (for GTPase measurements) or 0.3 mM NADH (for ATPase measurements), 32 µg/mL of lactate deshydrogenase, 60 µg/mL pyruvate kinase, and 4 mM GTP (for PatA/PatB) or 10 mM ATP (for BmrA). The buffer was heated at 37 °C during 5 min before adding the protein. Activities of 2 µg of PatA/PatB or BmrA (or 1 µg for BmrA proteoliposomes) were then measured by absorbance at 340 nm during 20 min at 37 °C. For measurements in detergent, LMNG was supplemented at 0.02% (PatA/PatB) or 0.01% (BmrA), while FC12 was supplemented at 0.15% in the cuvette.

## Supplementary information

Supplementary Information

## Data Availability

The datasets generated during and/or analyzed during the current study are available from the corresponding author on reasonable request.
