# Supplementary material for: Functionality of membrane proteins overexpressed and purified from E. coli is highly dependent upon the strain
Source: Sci Rep. 2019 Feb 25;9:2654. doi: 10.1038/s41598-019-39382-0 (PMC6390180; doi:10.1038/s41598-019-39382-0)
Supplement: Supplementary file 1 — Supplementary Information [file 41598_2019_39382_MOESM1_ESM.pdf]

Supporting Information

Functionality of membrane proteins overexpressed and purified from *E. coli* is highly dependent upon the strain

Khadija Mathieu[1], Waqas Javed[1,3], Sylvain Vallet[1], Christian Lesterlin[1], Marie-Pierre Candusso[1], Feng Ding[2], Xiaohong Nancy Xu[2], Christine Ebel[3], Jean-Michel Jault[1,*] and Cédric Orelle[1,*]

[1]Université de Lyon, CNRS, UMR 5086 "Molecular Microbiology and Structural Biochemistry", IBCP, 69367, Lyon, France

[2]Department of Chemistry & Biochemistry, Old Dominion University, Norfolk, VA 23529, USA

[3]Université Grenoble Alpes, CNRS, CEA, IBS, 38000, Grenoble, France

* Corresponding authors: cedric.orelle@ibcp.fr; jean-michel.jault@ibcp.fr; Phone : +33 (0)4 37 65 29 07 ; Address : 7 Passage du Vercors, 69367 Lyon Cedex 07, France

Table S1: purification yield (mg per L of culture) of PatA/PatB and BmrA transporters.

	LMNG	FC12
C41(DE3)-PatA/PatB	6.3	12
BL21(DE3)-PatA/PatB	2.1	11.8
C41(DE3)-BmrA	6.0	6.8
T7 express-BmrA	0.9	3.4

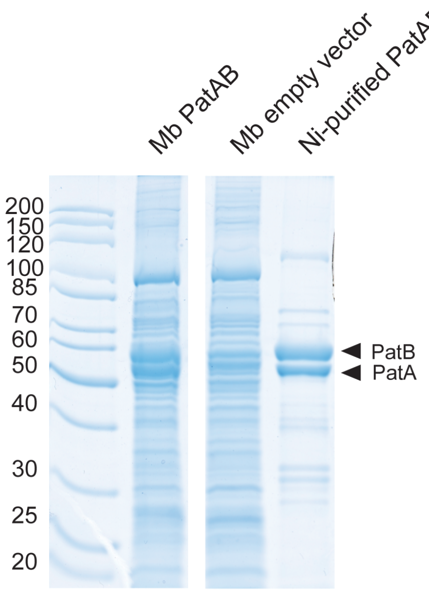

Figure S1. Membrane of C41(DE3) strain containing overexpressed PatA/PatB. A Coomassie-stained 12% SDS-PAGE was used to resolve the membrane fraction (Mb) of C41(DE3) strain expressing PatA/PatB, or not (empty vector control). On the right, PatA/PatB purified from Ni-affinity was loaded to confirm the identity of the bands.

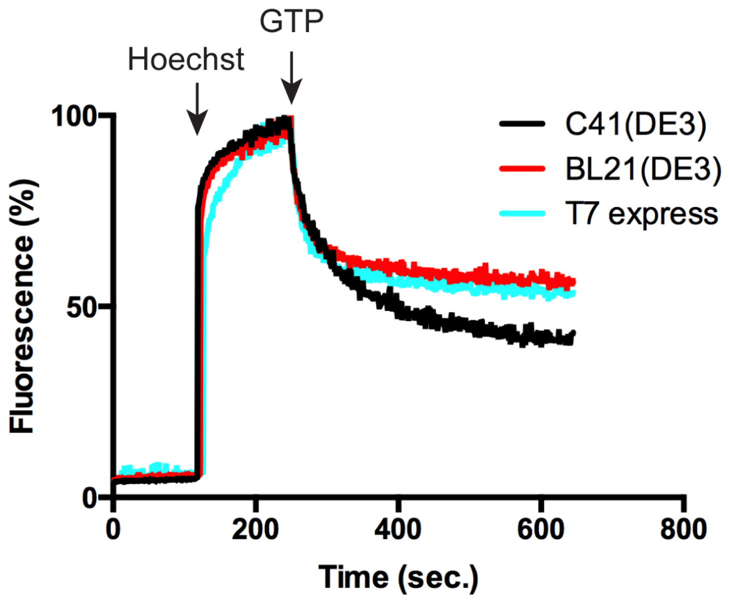

Figure S2. Hoechst 33342 transport assays with inverted membrane vesicles prepared from the various strains. The expression of PatA/PatB transporter was induced by IPTG in exponential phase for 5 h. Fifty µg of total membrane proteins were used for each assay.

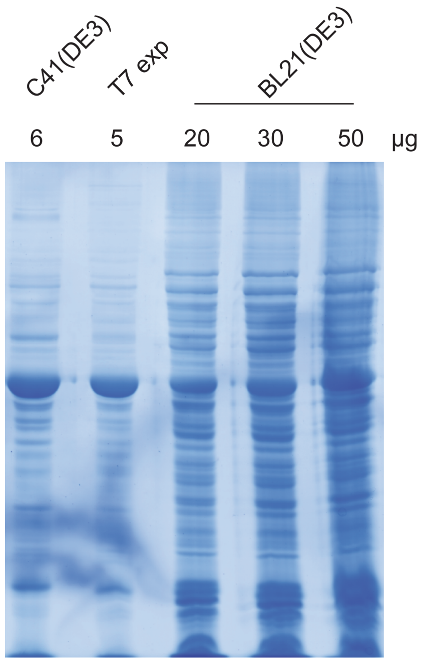

Figure S3. Approximate and relative quantification of BmrA in the different membrane preparations. BmrA appears to be about 10 times less expressed in BL21(DE3) strain as compared to the two other strains.

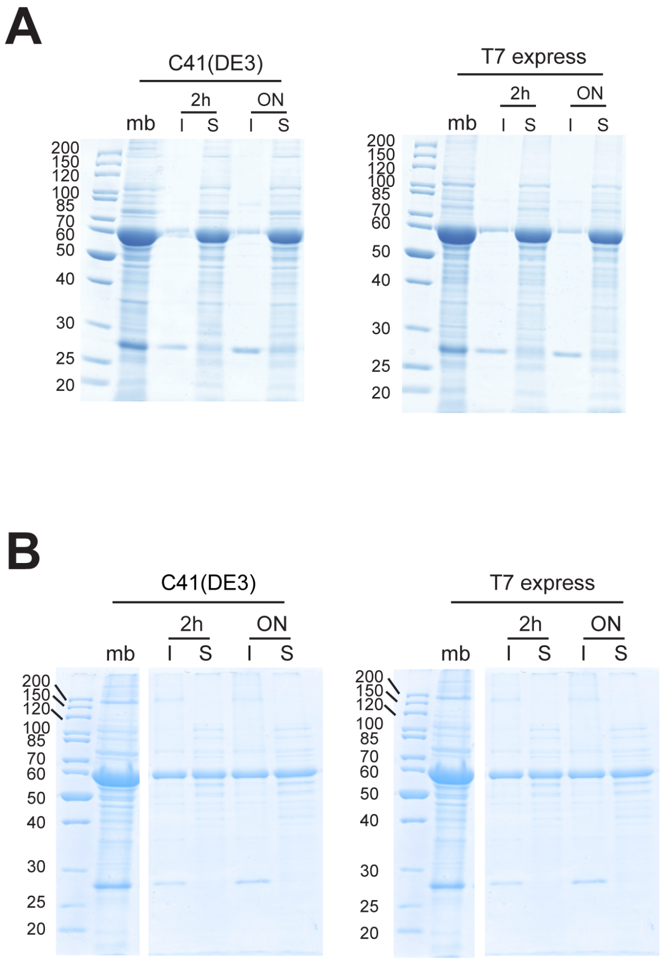

Figure S4. Solubilization of BmrA by Fos-Choline 12 or Triton X100. A, solubilization by Fos-Choline 12. **B**, solubilization by Triton X-100. Solubilization was performed either for 2 h or for overnight incubation. After ultracentrifugation, the soluble (S) and insoluble (I) fractions were analyzed by 12% SDS-PAGE.

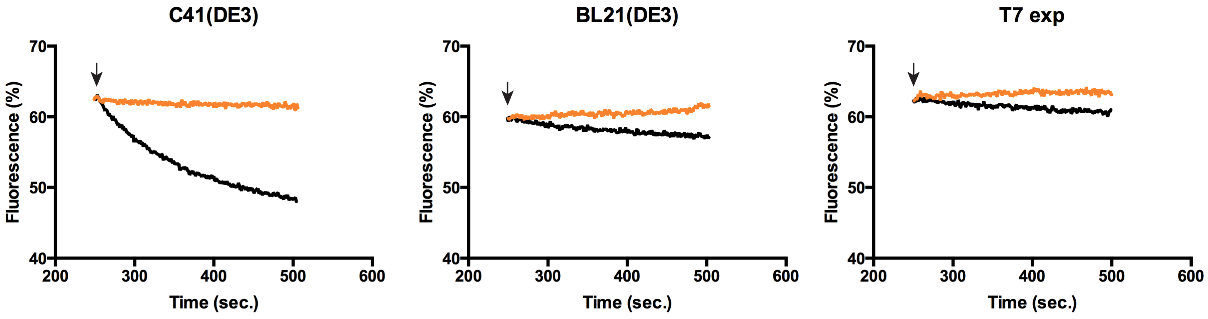

Figure S5. Doxorubicin transport assays with inverted membrane vesicles prepared from the various strains overexpressing BmrA-GFP. For the assays, 200 µg of total proteins were used and transport was monitored at 25 °C. Transport by membranes expressing BmrA-GFP is displayed as black traces, while transport by control membranes expressing the empty vector pET23 is displayed as orange traces.

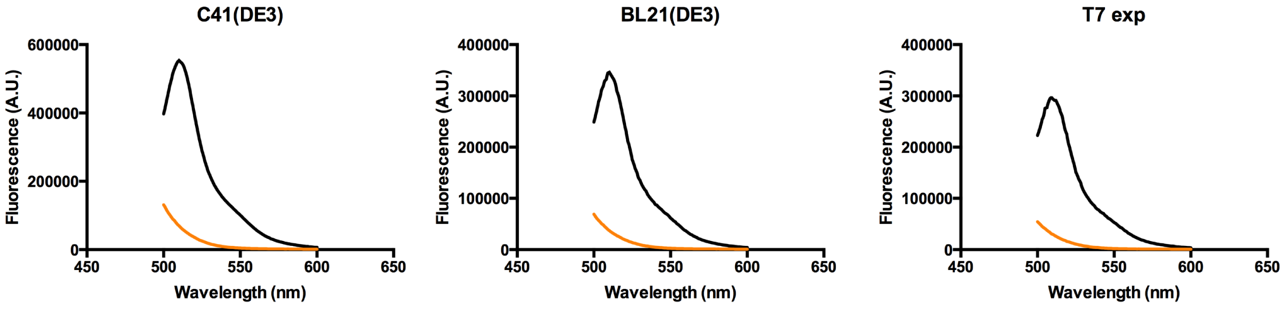

Figure S6. Quantification of GFP fluorescence in the membrane of the various strains. One hundred micrograms of total membranes were analyzed. Membranes expressing BmrA-GFP are displayed as black traces, while control membranes prepared with the empty vector pET23 are displayed as orange traces. Integrated fluorescence intensities were calculated between 500 and 530 nm. Background fluorescence calculated with control membranes represented respectively 13.5%, 11.6% and 10.6% of the total fluorescence in C41(DE3), BL21(DE3) and T7 exp, respectively.

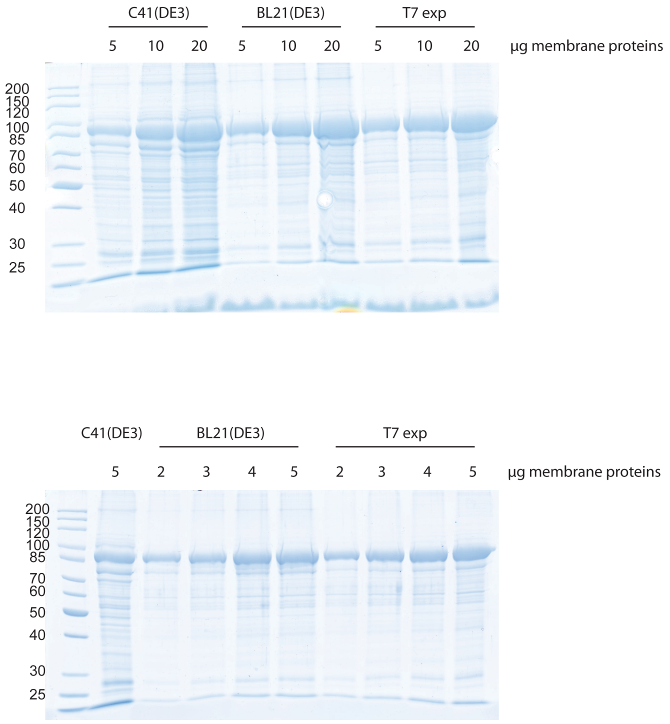

Figure S7. Relative quantification of BmrA-GFP in the membrane of the various strains. From the two gels, it was estimated that 5 µg of C41 membranes contained similar BmrA-GFP amounts than 3.5 µg of BL21 membranes or 3 µg of T7 express membranes.

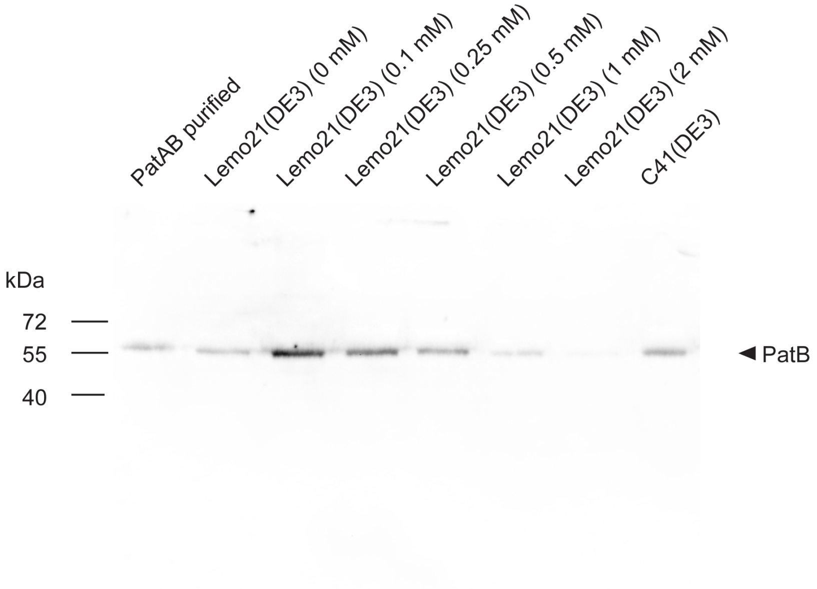

Figure S8. Western blot analysis of PatA/PatB expression in Lemo21(DE3) strain. An antibody anti his-tag was used and it revealed the his-tagged PatB subunit. Purified PatA/PatB was also loaded as a positive control. The experiment shows the highest expression in Lemo21(DE3) strain at 0.1 and 0.25 mM rhamnose. While 30 µg of membrane proteins were loaded for Lemo21(DE3) strain, 6 µg membrane proteins were loaded for C41(DE3) strain.

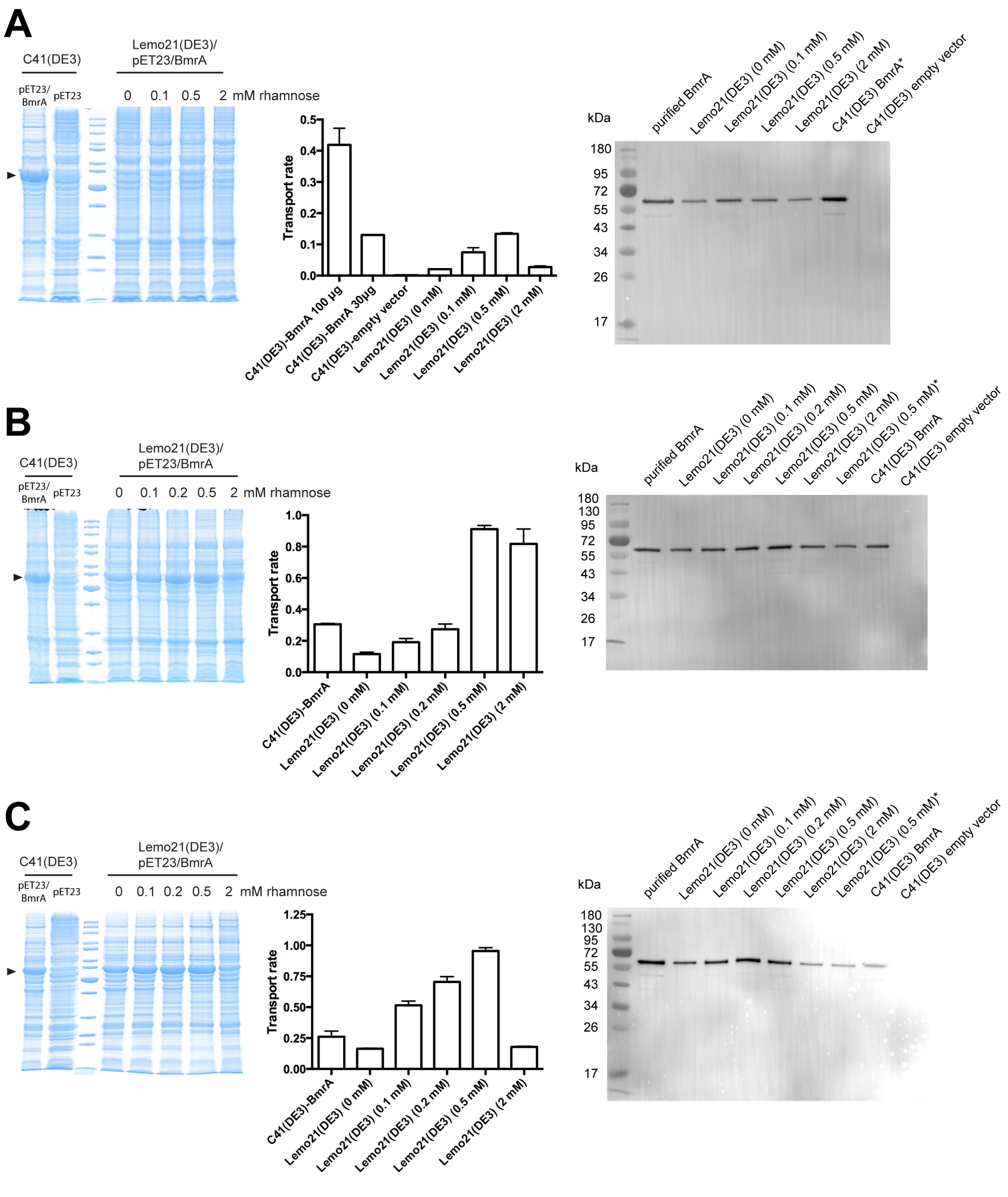

Figure S9. Expression and functionality of BmrA in membranes of C41(DE3) and Lemo21(DE3) strains. **A**, cells were induced in TB medium at 25 °C. Membrane fractions were analyzed by 12% SDS-PAGE. Transport rates were measured with 100 µg total membrane proteins. Transport displayed by the C41(DE3) membranes was ~3-4 times higher than the Lemo21(DE3) membranes prepared with 0.5 mM rhamnose. Western blot analysis was performed with 0.2 µg purified BmrA, 1 µg of membrane proteins (except for the lane with the *, which was loaded with 3 fold less membrane proteins to match the effective transport ratio). **B**, cells were induced in TB medium at 37 °C. Membrane fractions were analyzed by 12% SDS-PAGE. Transport rates were measured with 100 µg total membrane proteins. Transport displayed by the Lemo21(DE3) membranes prepared with 0.5 mM rhamnose was ~3 times higher than the C41(DE3) membranes.

Western blot analysis was performed with 0.2 µg purified BmrA, 0.3 µg of membrane proteins (except for the lane with the *, which was loaded with 0.1 µg, i.e. 3 fold less membrane proteins to match the effective transport ratio). Note that the intensity of BmrA from C41(DE3) is intermediate between the intensities of the bands in the Lemo21(DE3) at 0.5 mM rhamnose (see the 2 lanes with 0.5 mM), suggesting that BmrA overexpression is ~1.5 fold higher in this condition as compared to C41(DE3). **C**, cells were induced in LB medium at 37 °C. Membrane fractions were analyzed by 12% SDS-PAGE. Transport rates were measured with 100 µg total membrane proteins. Transport displayed by the Lemo21(DE3) membranes prepared with 0.5 mM rhamnose was ~3 times higher than the C41(DE3) membranes. Western blot analysis was performed with 0.2 µg purified BmrA, 0.3 µg of membrane proteins (except for the lane with the *, which was loaded with 3 fold less membrane proteins to match the effective transport ratio).

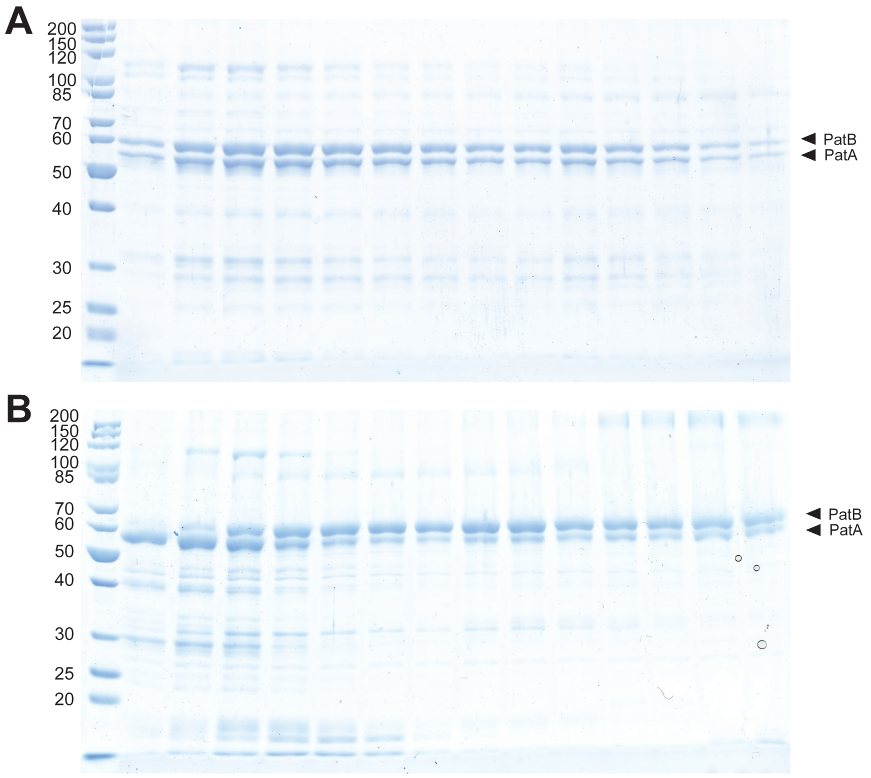

Figure S10. Elution profile of PatA/PatB after Ni-affinity purification. A, purification in 0.02% LMNG. PatA/PatB were eluted using a gradient of imidazole (fractions shown between 35 and 50 mM, from left to right). **B**, purification in 0.3% FC12. PatA/PatB were eluted using a gradient of imidazole (fractions shown between 60 and 80 mM, from left to right).
